# Haptic Feedback to Assist Blind People in Indoor Environment Using Vibration Patterns

**DOI:** 10.3390/s22010361

**Published:** 2022-01-04

**Authors:** Shah Khusro, Babar Shah, Inayat Khan, Sumayya Rahman

**Affiliations:** 1 Department of Computer Science, University of Peshawar, Peshawar 25120, Pakistan; khusro@uop.edu.pk (S.K.); sumayya2khan@gmail.com (S.R.); 2 College of Technological Innovation, Zayed University, Dubai 144534, United Arab Emirates; 3 Department of Computer Science, University of Buner, Buner 19290, Pakistan; inayat_khan@uop.edu.pk

**Keywords:** assistive technologies, indoor environment, smartphone, vibration patterns, contextual information

## Abstract

Feedback is one of the significant factors for the mental mapping of an environment. It is the communication of spatial information to blind people to perceive the surroundings. The assistive smartphone technologies deliver feedback for different activities using several feedback mediums, including voice, sonification and vibration. Researchers 0have proposed various solutions for conveying feedback messages to blind people using these mediums. Voice and sonification feedback are effective solutions to convey information. However, these solutions are not applicable in a noisy environment and may occupy the most important auditory sense. The privacy of a blind user can also be compromised with speech feedback. The vibration feedback could effectively be used as an alternative approach to these mediums. This paper proposes a real-time feedback system specifically designed for blind people to convey information to them based on vibration patterns. The proposed solution has been evaluated through an empirical study by collecting data from 24 blind people through a mixed-mode survey using a questionnaire. Results show the average recognition accuracy for 10 different vibration patterns are 90%, 82%, 75%, 87%, 65%, and 70%.

## 1. Introduction

Mental mapping of an environment is essential for orientation and mobility [1]. Most of the information is assembled visually by sighted people to perceive information [2]. In the absence of visual sense, demanding cognitive actions weaken [3]. Therefore, visual perception plays a dominant role in guiding people in an unknown environment and assisting them in performing their daily activities [4]. Unfortunately, people with visual disabilities lack this information and face several challenges while visiting different places, e.g., bus terminals, academic buildings, shopping malls, and offices. Therefore, they rely on other senses like touch, hearing, and haptic to explore these environments. Research on orientation and mobility in unknown spaces for blind people shows that spatial mapping and orientation skills should be endowed at two primary levels: perceptual and conceptual. Other senses like tactile, auditory, and haptic are rich sources for supplying spatial information at the perceptual level. The hand’s palm supplies haptic information and fingers to recognize object morph and texture. The auditory medium provides information about events in the environment, e.g., the presence of people, animals or machines, and distance information. At a conceptual level, the main focus is on developing suitable mapping and path generation strategies, e.g., walking along the room wall and exploring objects attached to the wall [5]. Thus, spatial information is delivered to blind people either through a conceptual or perceptual level, which assists them in performing their daily activities with a bit of ease by providing some sort of feedback. Over the last decade, significant efforts have been made to solve the issue of conveying non-visual information to disabled people in the form of feedback visually.

Feedback is the communication of spatial information to blind people for perceiving the surroundings [6]. It is used to properly guide blind people while navigating, keeping their capabilities, needs, and requirements in mind. The purpose of feedback is to help them to perform different tasks efficiently and safely, e.g., navigation, shopping, and interaction with their smartphone. Feedback should convey information related to a task to be understood by a user. Effective feedback communicates three things: the message, the situation, and the risk. People with visual disabilities have a high risk of injury or falling if they are unfamiliar with the environment. Therefore, feedback saves time, reduces frustration, and helps blind persons to focus on what they are doing correctly.

With progress on the assistive technologies of smartphones, it aims to deliver significant feedback to blind people for different activities like dialing a number, messaging, wayfinding, and exploring unknown environments [7]. A smartphone allows blind people to interact with ecosystems of services (home or shopping malls) using gestures, touch, auditory, and tactile [8,9]. In shopping malls [10], blind people can scan labels using their smartphone’s camera, guiding them through verbal instructions and telling them about the details, e.g., name, price, and manufacturer. 

Various smartphone-based approaches have been developed for blind people to perceive their surroundings and get feedback. These solutions are critically analyzed for their pros, limitations, and new opportunities. For a brief survey on recent work, we analyzed the previous surveys [11,12,13], which have provided a review of existing solutions considering various aspects. They identified several issues that have not been resolved while delivering information to visually impaired people and suggested modifying their interfaces to solve these accessibility issues. Bossini et al. [11] provided a set of guidelines resulting from reviewing the literature to be applied in a mobile applications context to access information by old and visually disabled people. Secondly, they have surveyed three native applications: Big Launcher, Fontrillo, and Mobile Accessibility for Android. These applications were analyzed and aimed to modify their interface to address accessibility issues, e.g., touch screen devices are not properly designed for them; developers do not address accessibility issues while designing interfaces. Harrison et al. [12] reviewed mobile usability and found that usability is usually measured by considering different attributes. Current research demonstrates that cognitive load is the most prominent attribute directly related to the success or failure of an application. They introduced the People at the Centre of Mobile Application Development (PACMAD) usability model by taking attributes from different models to overcome the limitation of existing models, e.g., Effectiveness, Efficiency, Satisfaction, Learnability, Memorability, Errors, and Cognitive load. After reviewing the literature, each attribute was found in at least 20% of studies. Developers sometimes ignore some issues: high power consumption, small screen size, limited connectivity, limited input modalities, and context. They explored that some researchers identified different attributes, but most of the existing models do not consider the context (environment) and cognitive load. Hakobyan et al. [13] reviewed and discussed different innovative applications and their information presentation methods specially designed for blind people. Most of the systems have used tactile and auditory-based information presentation methods as alternative approaches to traditional methods. These alternative modalities apply to specific applications like websites, charts, and graphs. They have reviewed several assistive technologies and concluded that particular mobility, orientation, and navigational capabilities of visually impaired people must be recognized, understood, and accommodated during the design phase of mobile assistive technologies.

With recent technological advances, different feedback mediums enable blind users to expand their knowledge. Several experimental approaches are carried out to use different mediums for effective feedback using a smartphone. The concerted efforts presented three types of feedback methods that are: voice [10,14,15], sonification [14,15], and vibration feedback [10,16,17] that ensure the significance of each feedback medium. In the absence of a visual medium, blind people typically use voice feedback to access information on the smartphone. Voice is direct communication that can deliver many messages to blind people. The latest smartphones offer accessibility services (e.g., Talkback and Voice over) for visually impaired users to interact with their surroundings. Researchers have used smartphone’ speakers, headphones, external devices, and screen reading software to generate voice feedbacks. However, this media is not efficient in the case of noisy environments and has a high level of disturbance. Furthermore, voice feedback is highly language-dependent as it is designed for a specialized language of a specific area [18]. Sonification feedback [19,20] are non-speech sounds (musical tones) used to convey visually impaired people’s feedback messages. Sonification feedback has the advantage of information privacy, but it is sometimes difficult to understand the message and cannot be heard in a noisy environment. An alternative approach that has been explored is based on the usage of haptic feedback [21]. Haptic feedback uses vibration patterns to convey information to a user. It uses a vibration motor/actuator for pattern generation, driven by an electronic circuit. Haptic/vibration feedback is widely used for notification systems and is especially useful when the user’s auditory and visual senses are occupied. It is language-independent, can work in a noisy environment, and does not have an information privacy problem [21,22], so it is potentially used as an alternative to speech and sonification feedback. Vibration feedback has significant advantages over speech [10,14] and sonification [19,20] feedback mediums.

We tend to apply smartphone vibration as a feedback medium. Nevertheless, there is still less knowledge of conveying information to blind people through haptic feedback for different tasks in indoor navigation. Semantic information can be conveyed to blind people via utilizing different vibration features, such as frequency, rhythm, and length. To identify the accurate vibration pattern using smartphone accelerometer sensors, we have used a frequency range from 2 Hz to 5 Hz. It is possible to generate vibration varieties, even with a single vibration motor, instead of using multiple external motors and hardware. However, designing patterns for every task will increase cognitive load, and the user will not recognize large varieties of patterns. 

Therefore, in this paper, we have categorized the blind people tasks in the form of taxonomy and incorporated them into a vibration pattern design. To increase the coverage of the vibration pattern, we have incorporated the natural-sound-like vibrations in combination with Morse code [20]. We have developed a real-time feedback system specially designed for blind people to convey information to them based on vibration patterns. Finally, the proposed solution has been evaluated through an empirical study by collecting data from 24 blind people through a mixed-mode survey using a questionnaire. Results show the average recognition accuracy for 10 different vibration patterns are 90%, 82%, 75%, 87%, 65%, and 70%.

## 2. Methods

**Figure 1:**
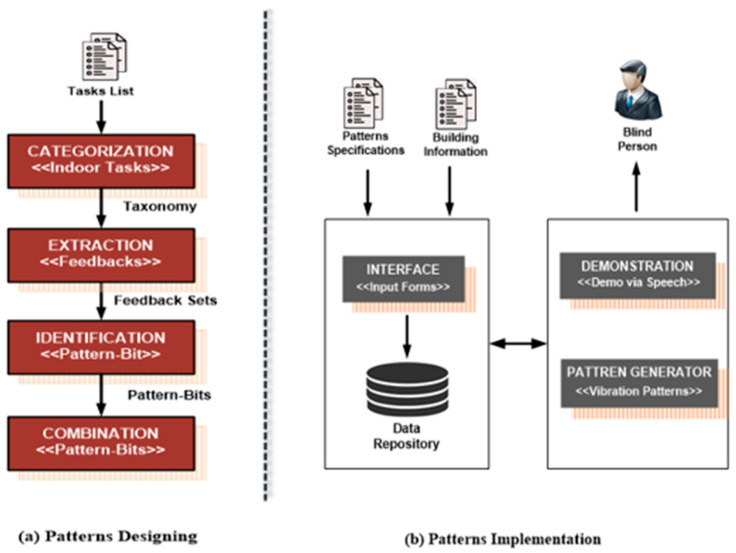
Overview of vibration patterns feedback for blind people.

A real-time feedback system has been developed to cope with the issues that address some of the aforementioned issues and assist blind users in navigation. The previous studies have demonstrated that vibration feedback contributes to the research of eyes-free interaction, as the vibration signals can complement existing interaction techniques [23]. Therefore, we have used different vibration patterns in our proposed solution. The proposed solution is achieved in 2 phases, i.e., the pattern designing and pattern implementation. To minimize the cognitive load, our fundamental step is to categorize the tasks based on similarity using the taxonomy approach, as shown in Figure 1a. First, we have developed a task taxonomy that provides abstract and concrete contextual information of each task. It will significantly reduce pattern varieties and ultimately balance the cognitive load. After categorization, the next step is pattern designing, where the vibration patterns are comprised of pattern-bits having appropriate pattern lengths.

We have designed different pattern-bit types that mimic the natural-sound representations (e.g., heartbeat, gun fire, etc.). We leverage the Morse code concept for the systematic arrangement of pattern-bits [24]. Pattern length varies for different tasks and depends on the task type and severity levels. In the implementation phase, we have developed a smartphone application, as shown in Figure 1b, that stores schematic information of vibration patterns and generates vibration patterns accordingly. The application also has a demonstration module to educate blind people with vibration patterns and their related feedback. The overall process of the proposed system is shown in Figure 1; further explanation of each step is given as follows:

### 2.1. Patterns Designing

To design a vibration pattern, our fundamental step is to identify blind people’s indoor tasks that may be navigational or have the risk of any danger and then categorize these tasks based on similarity using the taxonomy approach. The taxonomy is carried out in sequential steps of accumulating feedback messages: feedback phrases ◊ feedback ◊ feedback set, where the feedback set is the fundamental subject of the vibration pattern. After the extraction of the feedback sets, the next step is the identification of pattern-bits for designing patterns. So, we have developed different pattern-bit types that mimic the natural-tones representations (e.g., heartbeat, gun fire, etc.). After designing various bit-types, we systematically arranged pattern-bits with short, medium, and long lengths. For this purpose, we have used the concept of Morse code for pattern-bits systematic combination, which are easy to interpret and memorize. Each step of pattern designing is briefly discussed below.

The fundamental step of task categorization is to identify the tasks performed by blind people in a building that may be navigational or risky for them. For this purpose, our first step is to create a list of tasks categorized as: navigational tasks and risky tasks and then identify similarities among them. We consider academic buildings that visually impaired people mostly visit for task selection.

We finalized a list of navigational and risky tasks that are mandatory to instruct a blind person to be aware of the risks in an indoor environment, shown in Table 1. Navigational instructions include those instructions that have to do with directions (e.g., left, right, forward, and up), motion (hold, turn, walk and exit) and landmarks. Blind people meet various risks that are harmful and cause injuries to them. For this purpose, we selected those tasks which have risks of falling, collision, cut and burn, etc.

#### 2.1.1. Extraction of Feedback Sets

**Figure 2:**
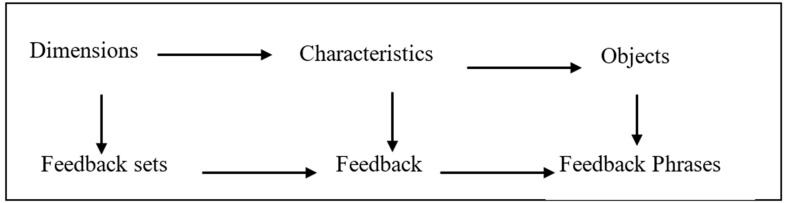
Classes of Taxonomy.

We have extracted different dimensions at the upper class of taxonomy, which are taken as “feedback sets” as they are categorized based on common characteristics. The characteristics are referred to as feedback, and the granular level objects are taken as feedback phrases. In the first iteration of taxonomy development, we have identified 2 main dimensions: Actionable and Contextual tasks. The first dimension, D1, has 2 common characteristics: actions that change direction and motion commands. The second dimension, D2, also has 2 common characteristics: tasks that have to do with architectural and environmental information. These characteristics were extracted from the granular level objects of taxonomy that have been identified from various sources, e.g., web surfing, environmental observations, and discussions with blind users. For designing vibration feedbacks, we named taxonomy classes as shown in Figure 2. 

So, after the first iteration, we have extracted 2 feedback sets, each having 2 feedbacks, and in the second iteration, we have observed that most of the tasks have the risk of injury, falling, collision, etc. Upward ramp, downward ramp, elevator status, upward elevator, downward elevator, upward escalator, downward escalator, upward stairs, downward stairs, in contact with blades, scissors, knife, broken glass, fire, and hot water. So, we have identified some common characteristics for these tasks, further grouped into 2 dimensions: Floor-changing and Severe-injury. The first dimension Floor-changing task, D3, has 2 common characteristics: manual and the automatic mean of changing the floor. Similarly, the second dimension Severe-injury, D4, has 2 common characteristics: cut and burn. 

**Figure 3:**
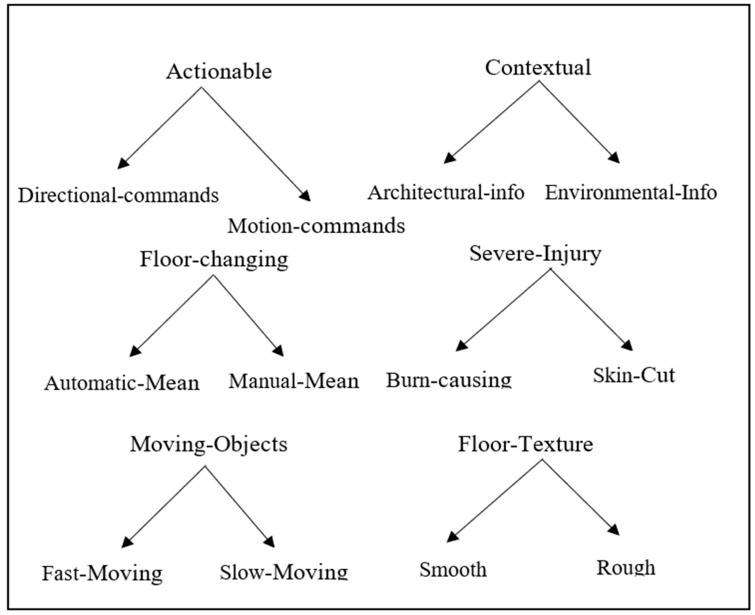
Feedback sets after the first, second, and third iteration.

In the third iteration, we used a conceptual-empirical approach where we initially identified some dimensions and then extracted their characteristics and instances. At this stage, we added 2 dimensions: D5, Moving-objects and Floor-texture having the same characteristics. Six different feedback sets after the first, second, and third iteration are depicted in Figure 3.

We have the following feedback sets with corresponding feedback (characteristics). 

Feedback Set1 = Actionable      Feedback1 = (Directional, Motional)

Feedback Set2 = Contextual      Feedback2 = (Architectural, Environment)

Feedback Set3 = Floor-change     Feedback3 = (Automatic, Manual)

Feedback Set4 = Severe-injury     Feedback4 = (Fire-Safety, Sharp cut)

Feedback Set5 = Moving objects     Feedback5 = (Fast, Slow)

Feedback Set6 = Floor-texture     Feedback6 = (Smooth, Rough)

The feedback sets are “the messages” that are the focal subjects of the vibration patterns.

#### 2.1.2. Identification of Pattern-Bits

After extraction feedback sets, our next step is to design a vibration pattern for the corresponding set. A vibration pattern is an arrangement of the repeatable alternating sequence of on and off states of a vibration motor, with specific lengths (short and long) assigned to each state. In order to produce unique repeatable sequences, an equal number of on and off signals must be alternated in arrangements that do not replicate any other sequence [22]. The vibration pattern comprises pattern-bits, the atomic unit of a pattern and has 2 properties: type and length. The type is used to represent the feedback set, and the length is used for the feedback in the set. We have designed different pattern-bit types that mimic the natural-sound representations. Natural sound-like representations are easy to interpret and memorize. 

**Pattern-bit 1(ms) = Heartbeat:** 0.50.150.50.700.50.150.50.1000.50.150.50.700.50.150.50

Duration = 3400 ms 

The vibration pattern with the above on-off state of the vibration actuator sounds like the lub-dub of a heartbeat. This length is taken as a pattern-bit, and in the next section, we have used different pattern-bit lengths and silence gape for the systematic arrangement using the concept of Morse code. After testing these patterns on the existing android application, we will decide which pattern is best suited for which 1 feedback set. 

**Pattern-bit 2 (ms) = Engine:** 125. 75.125.275.200.275. 125. 75. 125.275.20

Duration = 1695 ms

As the pattern’s name indicates, it sounds like starting an engine.

Pattern-bit 3 (ms) = Knock: 0.50.100.50.500.50.100.50

Duration = 900 ms

This pattern gives the feeling of knocking on the door. This will be easy to interpret as knocking is the most used activity.

**Pattern-bit 4 (ms) = Rapid/Hurry:** 0.50.100.50.100.50.100.50.100.50.100.50.100.50

Duration = 800 ms

This pattern gives the sensation of being in a hurry, so it can be used for those tasks which need immediate action. 

Pattern-bit 5 (ms) = Ringing-alarm: 0.1200.500.1200.500.1200

Duration = 4600 ms 

These two patterns give the feeling of hurry or urgency, so these are useful for urgent tasks.

Pattern-bit 6 (ms) = Down-stairs: 0.1000.800.700.500.400

Duration = 3400 ms 

This pattern sounds like someone is coming downstairs.

Pattern-bit 7 (ms) = Alert Buzzer: 0.500.250.500.250.500

Duration = 2000 ms

This pattern can alert the user to making a wrong decision. 

Some tasks require immediate action, so short lengthen patterns are used for immediate feedback. Similarly, medium, and long lengthen vibration patterns are used for normal feedback. The expected maximum feedback sets are 6 to 10, and one vibration pattern represents one feedback set. In the next section, we will systematically arrange these pattern-bits using Morse code.

#### 2.1.3. Accelerometer Calibration (for Vibration Frequency)

We have measured the performance of vibration frequency standards. In order to compensate for any smartphone variations of accelerometer sensor, the raw accelerometer data has been converted to a standard unit using device-specific parameters. The blind persons have been informed to hold their smartphones still and navigate towards different directions in the building. After, sufficient samples are collected for various activities including blind stationary mode, blind stairs down mode, left movement, right movement, etc. 

**Figure 4:**
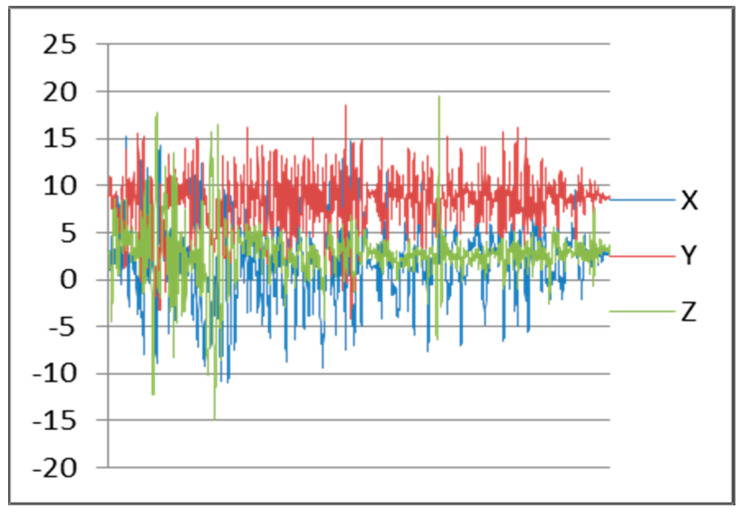
Downstairs Patterns.

**Figure 5:**
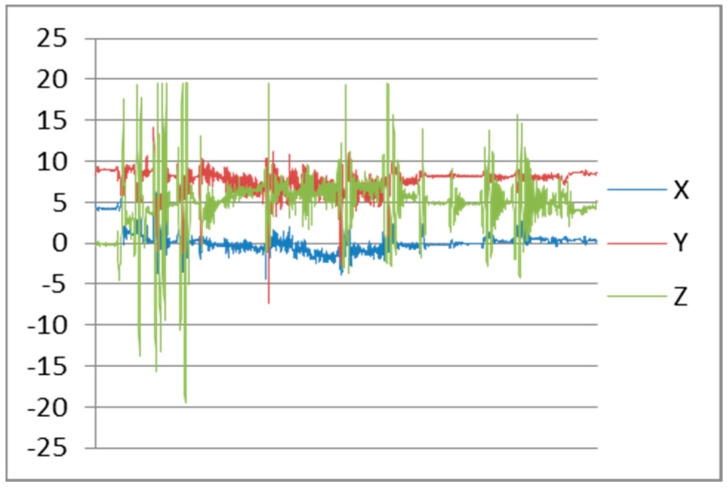
Left Movement Patterns.

**Figure 6:**
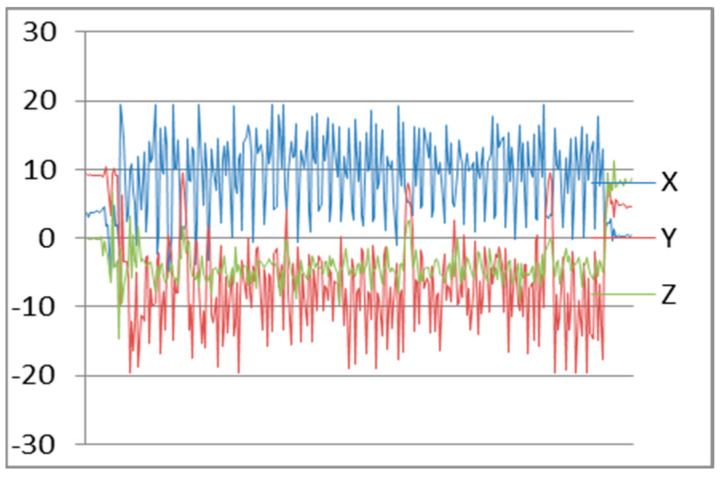
Walking Patterns.

After testing, we found the accelerometer sensor continuously reading data from x, y, and z axis’s. Maximum frequency of an accelerometer sensor can reach to 102 Hz. Frequency patterns for downstairs, left movement, and straight walking with smartphone in hand are shown in Figure 4, Figure 5, and Figure 6, respectively. 

#### 2.1.4. Combination of Patterns Components

**Figure 7:**
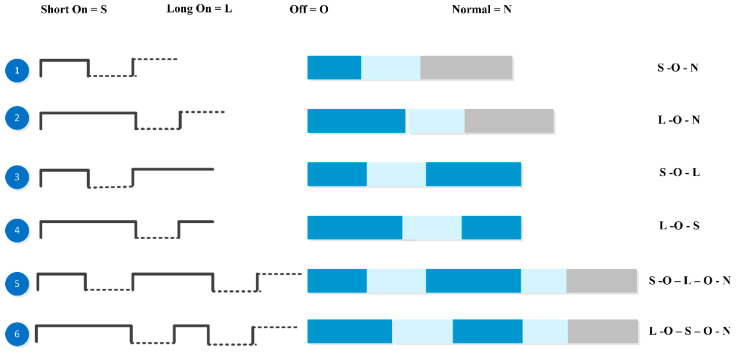
Patterns bit combination using Morse code.

We leveraged the Morse code concept for the systematic arrangement of pattern-bits [23]. Morse code is a method of conveying text information as a series of on-off tones, vibrations, or lights that a proficient listener or observer can directly understand. The International Morse Code encodes the ISO basic Latin alphabet and a small set of punctuation as standardized sequences of short and long signals called “dots” and “dashes,” as shown in Figure 7. Each text character is represented by a specific sequence of dots and dashes. The dash duration is 3 times longer than the dot duration. The dot duration is the basic unit of the code. We have used this method for arranging pattern-bits. A bit has 3 different lengths: short, medium, and long. Pattern-length is varied for different tasks and depends on the task type, and it is decided based on the scoring of urgency and risk level of activity. The length of the gap in pattern-bits and the duration of a bit affects a feedback message’s perceived level of urgency [22]. First of all, we have arranged each natural type pattern-bit with a combination of normal vibrations (600 ms) and designed up to 6 patterns for each natural type. Later on, we select the best-suited pattern for the corresponding feedback set after testing on the existing android app “Vibrate Tester.” Vibrating natural tone 1 time is taken as short on signal, and 2 times is taken as long on the signal. The silence gap is taken as 600 ms as below this value. It will be difficult to feel in case of these patterns. The following diagram shows a combination of pattern-bits. 

Here, we took Short on = 1-time natural tone, Long on signal = 2 times: similarly, Silence-off = 200 ms and Normal on = 600 ms.

We simply presented all the vibration patterns of each pattern-bit type sequentially and tested it so many times to rank them. After testing, we decided which 1 sequence must be chosen among these 6 patterns, e.g., for heartbeat bit type, we compared all patterns. Among them, pattern3 and pattern4 are more urgent than the other patterns as their total duration is short as compared to other patterns. Similarly, we compared the other bit type patterns and chose the best-suited one for corresponding feedback sets. The following example can easily explain it. In taxonomy, we have a class: Floor-Changing having feedback phrases like elevator, escalator, start, stop, downward, upward, and stairs at a granular level, which are then combined to form feedback. These feedback phrases are combined in 1 group as all of these are concerned with a task used to change the floor. So, for this task, we designed a floor-changing pattern as shown in Table 2. Pattern-Bit Type represents the feedback set, and Pattern-Bit length represents feedback in the feedback set.

### 2.2. Patterns Implementation

Finally, we have developed a smartphone application to implement and evaluate the vibration patterns. Each part of the implementation is discussed below:

#### 2.2.1. Designing User Interface

The application provides a form-based user interface to accept the designed schematic information of the vibration pattern and keep its related feedback set. The schematic information includes pattern name, pattern length, pattern-bit type, and duration, etc. The interface supports communicating instructions with haptic feedback. The interface communicates the instructions using different vibration patterns. The patterns differ in the type of pattern-bit in them and the length of these bits.

#### 2.2.2. Patterns Generation

We have simulated an academic building for pattern generation by storing its information in the data repository. The application persists the vibration patterns information for the feedback set and generates accordingly. For example, if the blind user is changing the floor, it generates its concerning pattern. 

#### 2.2.3. Patterns Demonstration

To educate the blind person, the application demonstrates the vibration patterns and their related feedback. We include a training module in this section to improve recognition accuracy for effective performance. The purpose of the training module is to train the user to use the patterns and is tested with generated patterns until they can memorize the different patterns.

## 3. Experimental Evaluation

To evaluate the accuracy, speed, and workload (cognitive load) of proposed vibration patterns, we first simulated an academic building and stored the building information in the application via a form-based user interface. Afterwards, we conducted a user study with different blind and low-vision participants groups. We had instructed the participants to interpret the patterns many times while walking in the building during the test. After completing the exercise of three months, the participants were asked to fill out the questionnaire to understand user satisfaction, perceived usability, user experience, and efficiency of the proposed solution.

### 3.1. Participants Recruitment

A total of 24 blind persons participated in this study. Among these, 20.83% (n = 5) were female and 79.16% (n = 19) were male subjects. The participants were filtered to have experience of using a smartphone for more than a year. Ages ranged from 19 to 49 and above years. Participants were categorized into four different age groups: 19–28 years (n = 13), 29–48 years (n = 06), 39–48 years (n = 04), and 49 and above (n = 01). Eight (n = 8) participants are totally blind and use the white cane in unfamiliar places. Five (n = 5) participants have been blind since birth and depend on a seeing-eye-dog for navigation assistance. Ten (n = 10) participants are visually impaired and particularly dependent on their cane for obstacles in the way. One (n = 1) is a 60 year old and needs human assistance for navigation purposes. Some of the participants are technology-oriented as they are regular users of the smartphone. The technologies adopted by the participants is shown in Table 3.

The main goals of the evaluation were to test the quality of the patterns provided in the testing session.

The testing took place at the Administration block of the Peshawar University campus. The building routes were simulated since the system is not integrated yet with the appropriate map representation. We chose that building because there are stairs, elevators, turns, tile, carpeted floor, and different obstacles. The users were asked to follow a trail route to get familiar with the tactile feedback. The user was asked to change Floor 1 to Floor 2 through stairs to start testing. Different obstacles were placed in the way to inform users using appropriate patterns for different tasks. The route included many intersections where the participant used the patterns to determine in which direction to continue walking and how to avoid obstacles. The modules first demonstrate the patterns to the user through voice feedback so that he/she may use it. Finally, we interviewed with a questionnaire to assess the mental load of using each vibration pattern, attitude towards usage of these patterns, etc. We briefly explained each method with each participant and conducted a lab test, a field test, and an interview. The observation i.e., reaction time, several times got a wrong turn, mental load, attitude towards the usage of vibration patterns, perceived usefulness, understandability and learnability, ease of use, system usability scale and user satisfaction were recorded in the testing session to test the quality of feedback.

### 3.2. Experimental Setup

A quantitative study was conducted to collect data from the participants for subsequent detailed analysis. For this purpose, participants completed a questionnaire in the form of a verbal interview about their preferences and opinions regarding the usage of vibration patterns for navigation in indoor environments. The questionnaire consisted of 40 questions that took approximately 20 min to complete. To simplify the process, questions in the questionnaire are simple propositions whose answers are selected from a 5-level Likert-Scale, ranging from “Definitely” to “Definitely Not”. The study was conducted individually, and group-wise, and the participants were informed about the types of questions, the purpose of data collection, the data collection procedure, and the evaluation system. 

To answer the query related to “Attitude towards the usage of vibration pattern in indoor navigation”, participants were asked four questions. For example, “Using a vibra-tion pattern is a good idea”, “I have a generally favorable attitude toward using the pro-posed solution”, “Overall, using a proposed solution is beneficial”, and “I think proposed solution makes my life more interesting”, To answer about Intention To Use (ITU), par-ticipants were asked four questions. For example, “I would recommend others to use the proposed solution”, “I predict I will use a proposed solution in the future as it makes it easy to understand vibration patterns”, “I plan to use a proposed solution in the future”, and “I expect my use of a proposed solution to continue in the future”. The participants were asked to answer the three questions in terms of Perceived Usefulness. For example, “Using the Proposed solution helped me in indoor navigation”, “I found the proposed solution unnecessarily complex“, and “I found the proposed solution easy to use”. Two questions were asked about Understandability and Learnability, e.g., “I found comfort and pleasure while learning and understanding the features”, and “I found feedback and haptic response conciseness in overall operations”. To answer query related “Easy-to-Use of the proposed solution”, participants were asked seven questions. For example, “It is easy to use”, “It is simple to use”, “It is user friendly”, “It requires the fewest steps possible to accomplish what I want to do with it”, “It is flexible”, “I can use it without verbal instructions”, and “I didn’t notice any inconsistencies as I use it”. Regarding the System Usability Scale, the participants were asked seven questions. For example, “I think that I would like to use this system frequently”, “I found the system unnecessarily complex”, “I think that I would need the support of a technical person to be able to use this system”, “I found the various functions in this system were well integrated”, “I found the system very cumbersome to use”, “I felt very confident using the system”, and “I needed to learn many things before I could get going with this system”. One of the main factors is the investigation of the mental workload when using the proposed vibration pattern. In this regard, the participants were asked eight questions. For example, “Are the abbreviations and acronyms used easy to interpret?” “Does it provide aids for entering hierarchic data?” “Is the guidance information always available?” “Does it provide hierarchic menus for sequential selection?” “Are selected data highlighted or covered with haptic responses?” “Does it indicate current position in menu structure?” “Are long data items partitioned?” and “Does it provide supplementary verbal labels for icons?” Finally, the user satisfaction about using the proposed vibration patterns has been estimated. In this regard, the participants were asked to answer the five questions. For example, “I am satisfied with it”, “I would recommend it to a friend”, “It works the way I want it to work”, “It is wonderful”, and ”It is pleasant to use”.

## 4. Results

The data collected from the questionnaire has been used for empirical analysis. In this study, we have performed different types of tests and analyzed the data using statistical tools like STATA and Excel. For better analysis and interpretations, we have performed descriptive tabulation by reporting the frequencies and percentages of the categories of the eight latent variables. To check the internal consistency and data reliability, we have measured the Cronbach of the latent variables. After analysis, we have proven that all the latent variables are internally consistent. Furthermore, we have performed a factor analysis in which Iterated Principal Factor analysis was found better. The purpose of these tests was to investigate the relationship between the latent variables, i.e., attitude, intention to use, perceived usefulness, understandability and learnability, ease of use, system usability scale, and user satisfaction.

### 4.1. Descriptive Tabulation

Participants’ responses show the descriptive statistics of frequencies and percentages of the categorical indicators of all latent variables. We have used eight latent variables in our study. 

**Figure 8:**
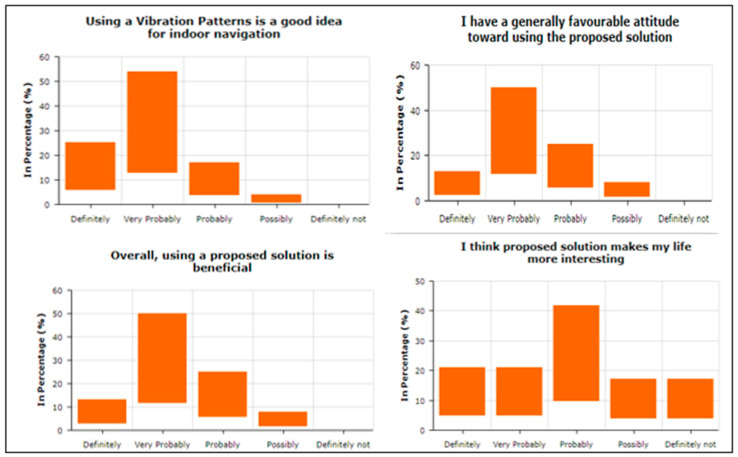
Attitude towards usage of the proposed solution.

**Figure 9:**
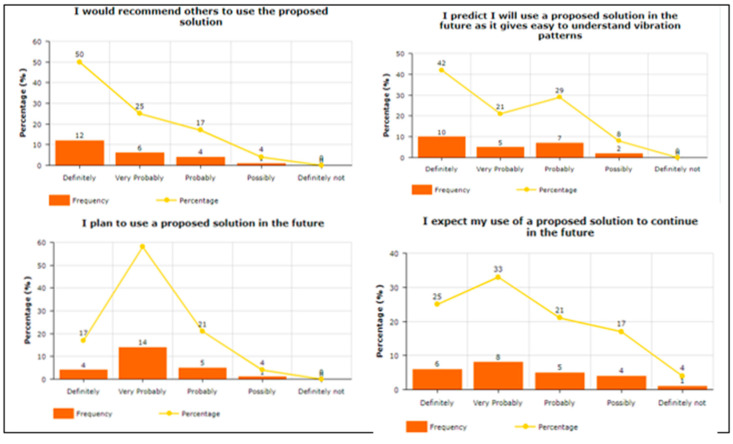
Intention to Use.

**Figure 10:**
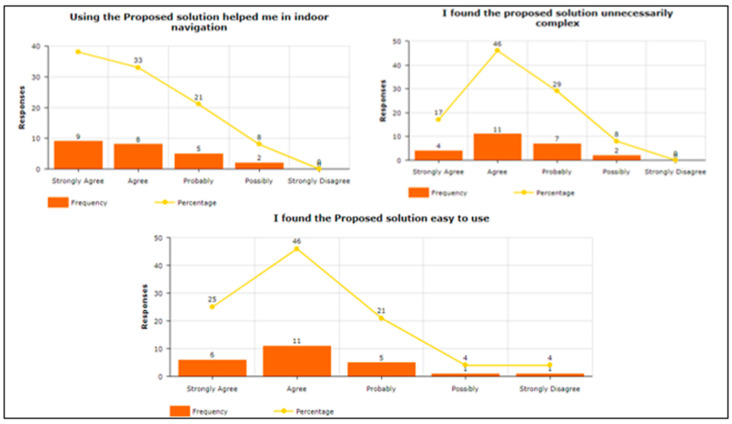
Perceived Usefulness after using the proposed vibration patterns.

As shown in Figure 8, the attitude towards the usage of a proposed solution was positive as most of the respondents selected scales 3, 4, and 5, which means that using proposed vibration patterns are good, beneficial, and interesting. A variable Intention to Use (IU) contains four measurement items depicted in Figure 9, showing higher scales, i.e., 4 and 5, which means that respondents are agreed to recommend the proposed solution to others and hope to be used in the future. The responses received from the responses about the perceived usefulness were impressive, as depicted in Figure 10. The responses show averagely higher scales, i.e., 3, 4, and 5, which means that the proposed solution helped the blind people in indoor navigation and found the solution less complex and easy to use.

**Figure 11:**
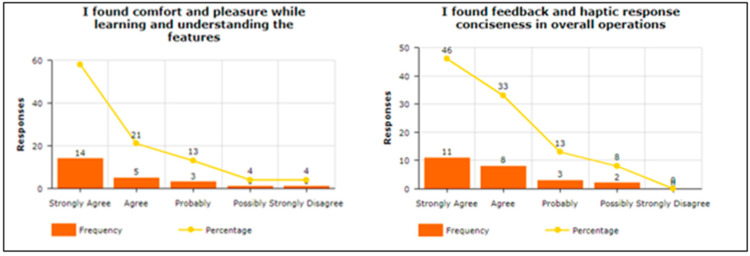
Understandability and Learnability.

The responses received from the participants regarding understandability and learnability were also impressive. As shown in Figure 11, higher scales have been used, which stated that the proposed solution is understandable and easy to learn its usage.

**Figure 12:**
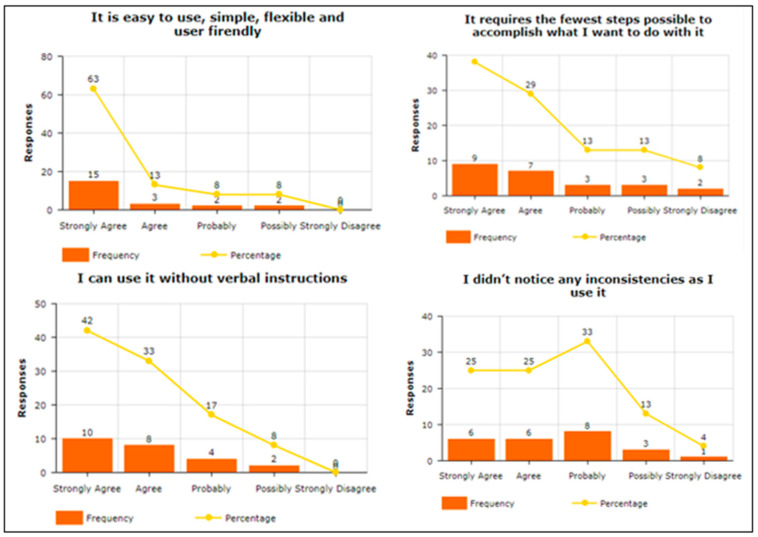
Ease of Use.

In terms of Ease of Use, as shown in Figure 12, a maximum number of participants have reported that the proposed solution is easy to use, simple, and flexible. They show higher scales for the questions that they will use the solution without verbal instructions as it requires very simple and fewer steps.

### 4.2. Factor Analysis

The Cronbach alpha test for measurement items (factors) has been conducted to check the reliability or internal consistency, as shown in Table 4. The alpha scores of all the factors are found reliable. We have reported principal component factor analysis (PCFA) as it gives us better results as compared to others. As shown in Table 5, the retained factors in PCFA show a clear contribution by a particular factor in total variation.

### 4.3. Experimental Results

**Figure 13:**
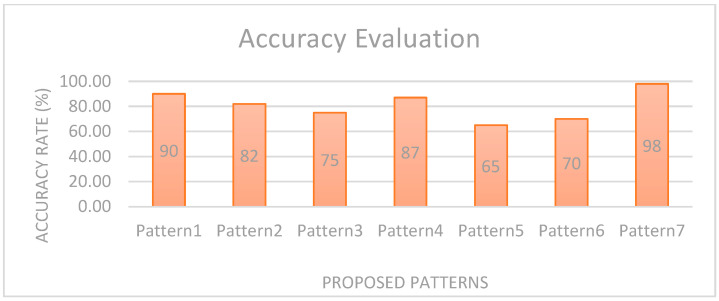
Recognition Accuracy among the proposed patterns after a field test.

The participants were explained the system and asked to continue to practice until they felt comfortable interpreting and memorizing the patterns for the respective feedback set. Participants said they could memorize up to 10 vibration patterns, and it is just a matter of familiarity. We would be able to memorize the different patterns with time. Most of the participants agreed that these patterns are easy to recognize as for each set, there is a different pattern-bit type. Secondly, the patterns sound like natural tones, which can be easily interpreted. They said that the heartbeat and door-knocking patterns are more familiar. P1 said that the pattern “Hurry” and “Engine” give the feeling of being hurried as they are short. There is only a little confusion for feedback in feedback sets as the pattern for feedback has the same bit type but differs in bit arrangement. This will be solved with time after practicing many times. Figure 13 shows the accuracy rate for the proposed vibration pattern recognition. The average recognition accuracy is 90%, 82%, 75%, 87%, 65%, and 70%. From this graph, we evaluated that Pattern1 and Pattern2 have more recognition accuracy as they are simple to recognize. Pattern5 has a 65% recognition rate as some participants had an issue recognizing it.

**Figure 14:**
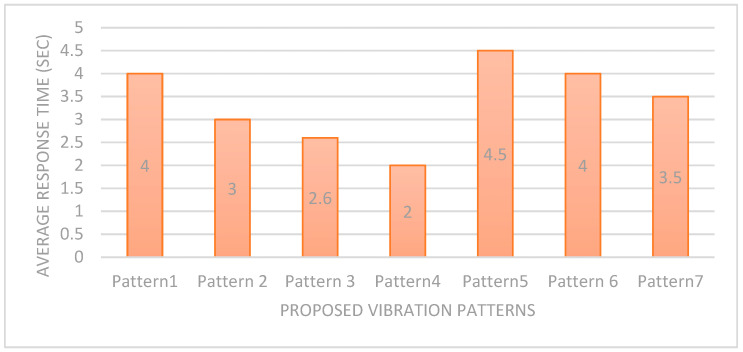
Average Reaction time per action of the user.

The next aspect of evaluating our feedback system is the average response time for action on feeling the pattern. Figure 14 calculated the average response time per action for each vibration pattern. The results indicate that Pattern1, Pattern5, and Pattern6 have the longest average response time (4 and 4.5 s). This is because the duration/length of these patterns is comparatively long; therefore, this takes a little bit more time to identify. Due to this reason, we have used these patterns for normal tasks. Similarly, Pattern3 and Pattern4 have less response time (2.9 and 3 s) as they have the shortest lengths. Therefore, they have been used for urgent and risky tasks. The average response time is the key factor in feedback, so it must not be that much longer.

**Figure 15:**
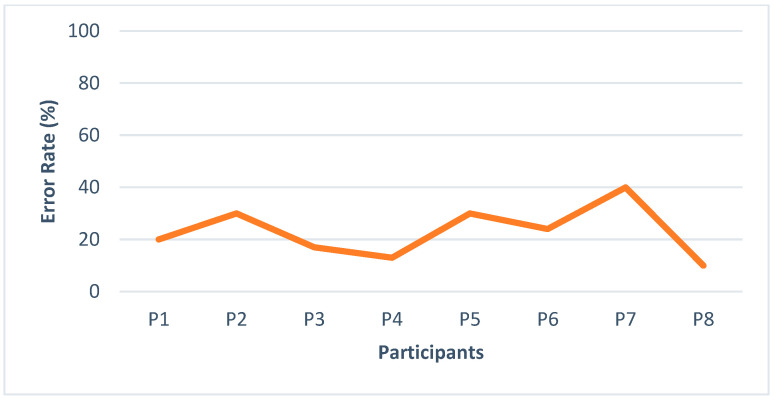
Errors occurring by participants while recognition.

Figure 15 shows the number of errors that occur with every participant while navigating in the building. As participants were regular users of the smartphone, they felt more comfortable with this feedback having an error rate of 17%, 20%, and 15%, respectively. Some participants took the wrong decision while taking a turn and coming downstairs, having an error rate. Pattern7 is used to inform the user of taking the wrong decision, e.g., deviation from route or upstairs instead of taking the elevator as the proposed system is a feedback module that can be further embedded with navigation systems so most of the modules can be integrated over there. One participant with a maximum age has never used a smartphone. His error rate was approximately 40%, and he suggested using voice feedback in combination with vibration for better efficiency.

## 5. Discussion

In recent years, vibration has been explored as an effective alternative feedback medium to voice and sonification for perceiving an environment by blind people. Vibration feedback can be used to convey real-time information with privacy control. However, the extensive use of vibration maximizes the cognitive load of a blind user when vibration varieties are large in number and because the gap between blind person activities and the usability of vibration patterns is not covered. Moreover, most of the systems have used external vibration motors and hardware to communicate only directional information. These problems need to be addressed to minimize the cognitive load of a blind user without using external hardware. We have proposed a vibration-based haptic feedback solution to assist blind people in indoor navigation to cope with these issues.

The proposed solution has been evaluated through empirical evaluation, where we have reported statistical tabulation along with factor analysis. We have used eight latent variables: Attitude, Intention to Use, Perceived Usefulness, Understandability and Learnability, and Ease of Use. Attitude towards the proposed solution was positive as most of the participants selected the high scales. Considering the Intention to Use, respondents showed higher scales, i.e., 4 and 5, which means that respondents are agreed to recommend the proposed solution to others and hope to be used in the future. Similarly, the responses received from the responses about the perceived usefulness were impressive. In summary, according to our obtained results, all of the eight variables are found satisfied. 

Furthermore, the Cronbach alpha test for measurement items (factors) has been conducted to check the reliability or internal consistency. The alpha scores of all the factors are found reliable. We have reported principal component factor analysis (PCFA) as it gives us better results as compared to others. The retained factors in PCFA show a clear contribution by a particular factor in total variation.

## 6. Conclusions and Future Work

With progress on the assistive technologies of smartphones, it aims to deliver significant feedback to blind people for different activities like dialing a number, messaging, wayfinding, and exploring unknown environments. Researchers have proposed various solutions for conveying feedback messages to blind people using these mediums. Voice and sonification feedback are effective solutions to convey information. However, these solutions are not applicable in a noisy environment and may occupy the most important auditory sense. The privacy of a blind user can also be compromised with speech feedback. We have proposed a tactile feedback solution to navigate blind people in indoor environments. We have used the accelerometer sensors to identify the vibration patterns for indoor navigation. We have classified blind navigation in different taxonomy and associated each vibration pattern. Hence, in order to make the proposed solution practical, we have adjusted the vibrating strength of the smartphone and calibrated the accelerometer sensor using gold standard as mentioned [25]. The focus of this work is to overcome the issues of existing feedback systems to facilitate blind users and provide more flexibility. Speech feedback could be optimal to convey messages to blind people but cannot be applied in a noisy environment and is also language-dependent. Therefore, tactile feedback is the most promising alternative approach to the speech feedback medium, but it has maximized cognitive load as the vibration varieties are large in number. Similarly, we have categorized blind people’s indoor tasks in the form of taxonomy based on similarity and designed natural vibration patterns for each group to the minimized cognitive load of blind users. While conducting field tests with different groups of blind people, it is observed that the proposed system outperforms traditional approaches such as speech, sonification, and simple vibration. Our proposed solution is only applicable in an indoor environment. However, it is not feasible to be used in an outdoor environment.

In the future, further research should be conducted to identify more tasks that may be outdoors as we have specifically extracted indoor tasks. We will further explore integrating this module into different navigation aids/applications for people with visual impairment. We will develop a real-time mobile application for visually-impaired people upon this tactile feedback module in future work.

## Figures and Tables

**Table 1:** Tasks performed by blind people in an indoor environment.

S. No	Navigational Task	Risky Task	Task Description
1	Walk Forward	Upward ramp	Fall
2	Walk Backward	Downward ramp	Fall
3	Turn Left	Upward Elevator	Fall
4	Turn Right	Downward Elevator	Fall
5	Turn Around	Elevator Door	Fall
6	Hold this Side	Upward Escalator	Fall
7	Forward	Downward Escalator	Fall
8	Enter	Upward Stairs	Fall
9	Exit	Downward Stairs	Fall
10	Stop	Rout Deviation	Fall
11	Enter	In contact with sharp things (e.g., blades, scissors, knife, and glass)	Cut
12	Exit	Fire Safety Risk	Burn causing
13	Stop	Hot water	Burn causing
14	Corridors	Hot iron	Burn causing
15	Door (sliding or push)	Obstacles in front	Collision
16	Destination	Slippery/wet floor	Fall
17	Landmarks	Smooth tiles	Fall
18	Floor #	Fast Moving object	Hit

**Table 2:** Pattern for Floor-changing feedback set.

	Property	Value
1	Pattern Name	Floor-Changing
2	Number of Pattern-Bits	Three
3	Pattern-Bit Type	Stairs down
4	Pattern-Bit Length	[short, silence, normal]
5	Pattern-Bit Duration	[3400 ms, 200 ms, 600 ms]
6	Vibration Pattern	[Stairs down—Silence gap—Normal]
7	Pattern Duration	3400 + 200 + 600 = 4200 ms

**Table 3:** Technologies adopted by the participants.

Technology	Q Mobile Linq l15	iPhone	Samsung Galaxy S5	Regular Phone	RP01 GPS Tracker
Participants	N = 04	N = 02	N = 12	N = 04	N = 02

**Table 4:** Data reliability test (Cronbach alpha).

Measurement Items	Observations	Item-Test Correlation	Item-Rest Correlation	Average Inter-Item Correlation	Cronbach Alpha
AT1	24	0.2520	0.4809	0.0351	0.8081
AT2	24	0.3014	0.0067	0.0431	0.8441
AT3	24	0.4815	0.1911	0.0402	0.8305
AT4	24	0.5102	0.1173	0.0494	0.8360
ITU1	24	0.6879	0.5205	0.0303	0.8050
ITU2	24	0.4606	0.2741	0.0394	0.8242
ITU3	24	0.5314	0.2433	0.0938	0.8266
ITU4	24	0.2702	0.0763	0.0143	0.8390
PDU1	24	0.3653	0.0714	0.0452	0.8394
PDU2	24	0.2956	0.0009	0.0436	0.8445
PDU3	24	0.0394	0.1474	0.0455	0.8338
UAL1	24	0.5501	−0.0446	0.0494	0.8478
UAL2	24	0.0147	0.2258	0.0353	0.8279
EU1	24	0.1644	0.2782	0.0395	0.8239
EU2	24	0.2261	0.3439	0.0313	0.8189
EU3	24	0.3280	0.3460	0.0313	0.8187
EU4	24	0.1534	0.1618	0.0432	0.8327
EU5	24	0.3241	0.3418	0.0312	0.8190
EU6	24	0.4337	0.1415	0.0463	0.8342
EU7	24	0.5384	0.0441	0.0484	0.8414
SUS1	24	0.4184	0.1258	0.0485	0.8354
SUS2	24	0.3973	0.0026	0.0432	0.8444
SUS3	24	0.4179	0.2291	0.0393	0.8276
SUS4	24	0.0104	0.0158	0.0421	0.8434
SUS5	24	0.3324	−0.0623	0.0432	0.8491
SUS6	24	0.3636	0.0697	0.0413	0.8395
SUS7	24	0.4689	−0.0259	0.0422	0.8464

**Table 5:** Principal Component Factor Analysis.

Measurement Items	Factor	Eigen Value	Variable	Factors			Uniqueness
				Fact1	Fact2	Fact3	
**Attitude**	Fact 1	0.5114	AT1	0.6211	0.1612	-	0.2116
	Fact 2	1.2723	AT2	0.8123	−0.5745		0.2145
	Fact 3	0.9015	AT3	0.4211	0.7034		0.5433
	Fact 4	0.5035	AT4	0.5122	−0.2446		0.3311
**Intention to Use**	Fact 1	1.3334	ITU1	0.6235	0.6673	-	0.4509
	Fact 2	1.1622	ITU2	0.6835	−0.4034		0.6307
	Fact 3	0.7835	ITU3	0.2853	0.6877		0.1244
	Fact4	0.7154	ITU4	0.6355	−0.4847		0.4745
**Ease of Use**	Fact 1	1.6714	EU1	−0.2723	0.1357	0.7911	0.2534
	Fact 2	1.3124	EU2	0.6845	−0.3524	0.0923	0.1122
	Fact 3	1.0615	EU3	0.0552	0.5634	0.0822	0.5643
	Fact 4	0.9711	EU4	−0.4455	0.9245	−0.2634	0.1912
	Fact 5	0.7913	EU5	0.8062	0.1345	0.1522	0.4412
	Fact 6	0.6545	EU6	−0.5224	−0.6547	0.1834	0.3666
	Fact 7	0.5134	EU7	0.0145	0.4877	0.5355	0.4756
**System Usability Scale**	Fact 1	1.4013	SUS1	0.7867	0.1279	0.0756	0.2233
	Fact 2	1.3145	SUS2	0.6068	0.1878	−0.1865	0.1936
	Fact 3	1.1514	SUS3	0.4589	−0.0178	−0.6744	0.1146
	Fact 4	1.0853	SUS4	0.0790	0.0554	0.1134	0.8874
	Fact 5	0.8453	SUS5	0.1887	0.6964	0.3456	0.1445
	Fact 6	0.6813	SUS6	0.2767	−0.6065	0.2144	0.1955
	Fact 7	0.5243	SUS7	0.3478	0.2288	0.6855	0.1466

## Data Availability

Data and materials are available on request.
